# Lipid-Based Nanoformulations for Drug Delivery: An Ongoing Perspective

**DOI:** 10.3390/pharmaceutics16111376

**Published:** 2024-10-26

**Authors:** Mubashar Rehman, Nayab Tahir, Muhammad Farhan Sohail, Muhammad Usman Qadri, Sofia O. D. Duarte, Pedro Brandão, Teresa Esteves, Ibrahim Javed, Pedro Fonte

**Affiliations:** 1 Department of Pharmacy, Faculty of Biological Sciences, Quaid-i-Azam University, Islamabad 45320, Pakistan; mrehman@qau.edu.pk; 2 College of Pharmacy, University of Sargodha, Sargodha 40100, Pakistan; ntahir1@mgh.harvard.edu; 3 Wellman Center of Photomedicine, Massachusetts General Hospital, Harvard Medical School, Boston, MA 02114, USA; 4 Department of Pharmacy, University of South Asia, Lahore 54000, Pakistan; farmacist.pk@gmail.com; 5 Department of Pharmacy, Faculty of Health and Medical Sciences, The University of Copenhagen, 1172 København, Denmark; 6 Australian Institute for Bioengineering and Nanotechnology, The University of Queensland, Brisbane, QLD 4072, Australia; m.munir@qu.edu.pk (M.U.Q.); i.javed@uq.edu.au (I.J.); 7 Department of Bioengineering, iBB-Institute for Bioengineering and Biosciences, Instituto Superior Técnico, University of Lisboa, Av. Rovisco Pais, 1049-001 Lisbon, Portugal; sofia.duarte@tecnico.ulisboa.pt (S.O.D.D.); pbrandao@egasmoniz.edu.pt (P.B.); teresa.esteves@tecnico.ulisboa.pt (T.E.); 8 Associate Laboratory i4HB, Institute for Health and Bio-Economy, Instituto Superior Técnico, University of Lisboa, Av. Rovisco Pais, 1049-001 Lisbon, Portugal; 9 Egas Moniz Center for Interdisciplinary Research (CiiEM), Egas Moniz School of Health & Science, 2829-511 Almada, Portugal; 10 Departamento de Química, Centro de Química de Coimbra-Institute of Molecular Sciences (CQC-IMS), Faculdade de Ciências e Tecnologia, University of Coimbra, 3004-535 Coimbra, Portugal; 11 Center for Marine Sciences (CCMAR), University of Algarve, Gambelas Campus, 8005-139 Faro, Portugal; 12 Department of Chemistry and Pharmacy, Faculty of Sciences and Technology, University of Algarve, Gambelas Campus, 8005-139 Faro, Portugal

**Keywords:** liposomes, SLN, targeting, controlled release, solubility enhancement, vaccine, hydrophobic, peptide, self-emulsifying

## Abstract

Oils and lipids help make water-insoluble drugs soluble by dispersing them in an aqueous medium with the help of a surfactant and enabling their absorption across the gut barrier. The emergence of microemulsions (thermodynamically stable), nanoemulsions (kinetically stable), and self-emulsifying drug delivery systems added unique characteristics that make them suitable for prolonged storage and controlled release. In the 1990s, solid-phase lipids were introduced to reduce drug leakage from nanoparticles and prolong drug release. Manipulating the structure of emulsions and solid lipid nanoparticles has enabled multifunctional nanoparticles and the loading of therapeutic macromolecules such as proteins, nucleic acid, vaccines, etc. Phospholipids and surfactants with a well-defined polar head and carbon chain have been used to prepare bilayer vesicles known as liposomes and niosomes, respectively. The increasing knowledge of targeting ligands and external factors to gain control over pharmacokinetics and the ever-increasing number of synthetic lipids are expected to make lipid nanoparticles and vesicular systems a preferred choice for the encapsulation and targeted delivery of therapeutic agents. This review discusses different lipids and oil-based nanoparticulate systems for the delivery of water-insoluble drugs. The salient features of each system are highlighted, and special emphasis is given to studies that compare them.

## 1. Introduction

The oral route for drug administration is a simple, naturally preferred, and reliable practice for treating most diseases [1,2]. However, oral delivery of lipophilic drug molecules is limited due to their low water solubility. Approximately 40% of new drugs display low aqueous solubility, exhibiting low dissolution, low drug absorption, high subject variability, degradation in biologically relevant media, and lack of dose proportionality [3,4]. Initially, the physicochemical properties of the drugs were modified for solubility enhancement by using techniques such as salt formation and particle size reduction [5]. Nevertheless, some drawbacks are associated with these methods, as neutral compounds are neither easily converted to salts nor made into a weak acid or weak base. It is also observed that the salt form may regain its original acid or base form in the gastrointestinal (GI) tract, reducing solubility [6]. Poor wettability, difficult handling, and particle size reduction may not be an issue in the case of very fine powders. 

Oils are used traditionally in the food industry for various purposes, one of which is to enhance the solubility of water-insoluble bioactive compounds. For example, a spoonful of oil may be added to a salad to improve the absorption of vitamin E, a lipophilic vitamin [7]. For decades, oils have been employed to enhance the solubility of lipophilic compounds. Sandimmune is a cyclosporine formulation that dissolves the drug in corn oil for solubility enhancement. Although later studies showed that the efficacy of Sandimmune is optimum in the presence of bile salts, which may act as surfactants, the enhanced bioavailability was due to the formation of tiny oil droplets (emulsion) absorbed from the GI tract [7,8]. The availability of different natural oils and fats and their synthetically modified derivatives helps to develop formulations with diverse compositions and physicochemical properties to achieve the optimal therapeutic efficacy of different therapeutic moieties [9]. Although emulsions are primarily investigated for oral or topical delivery, the development of lipid vesicles and solid-phase lipid nanoparticles has enabled intravenous or intramuscular administrations. The novel nanoparticles are versatile enough to load sensitive macromolecules or conjugate ligands on their surface.

In the last thirty years, there has been a significant increase in research on lipid nanocarriers. This has led to the development of various delivery systems with adjustable structure, desirable composition, and multifunctional properties. Lipid-based nanoformulations have seen significant clinical applications, particularly in drug delivery, due to their ability to enhance the bioavailability and targeting of therapeutic agents. For instance, liposomes, one of the most well-established lipid-based carriers, have been successfully utilized in products such as *Doxil* and *AmBisome*, which are used in cancer therapy and antifungal treatments, respectively. Solid lipid nanoparticles (SLNs) and nanostructured lipid carriers (NLCs) have also made their way to the market, with applications ranging from dermatological treatments (e.g., *Nanobase*) to more complex drug delivery systems. The recent success of lipid nanoparticles (LNPs) in mRNA-based COVID-19 vaccines, including Pfizer-BioNTech’s *Comirnaty* and Moderna’s *mRNA-1273*, further highlights the commercial and therapeutic potential of lipid-based systems. These formulations not only enable efficient encapsulation and delivery of hydrophobic drugs but also enhance stability, making them attractive for various routes of administration, including intravenous and oral. With ongoing research, the pipeline of clinical products based on lipid-based nanoformulations is expected to expand across a wide range of therapeutic areas.

This review highlights current trends in the development of different lipid nanocarriers, with a prime focus on their preparation and application in different clinical domains. It focuses on current trends in lipid nanocarrier research, and a special emphasis has been placed on comparing the effectiveness of these systems, highlighting their main advantages and the current challenges in this field that need further investigation to optimize them for clinical translation.

## 2. Micro- and Nanoemulsions

Lipids are widely studied as a tool to enhance the solubility and bioavailability of water-insoluble drugs classified in biopharmaceutical classes II and IV [10]. The most appreciated strategy is the addition of a lipophilic drug into inert lipid vehicles, such as oils, to form nano-scale drug delivery systems (DDS) like emulsions (also including microemulsions and nanoemulsions) and self-emulsifying drug delivery systems (SEDDS). These lipid-based dispersion systems often synergize the encapsulation and release of lipophilic and hydrophilic compounds with various physicochemical properties.

### 2.1. Microemulsions

**Figure 1:**
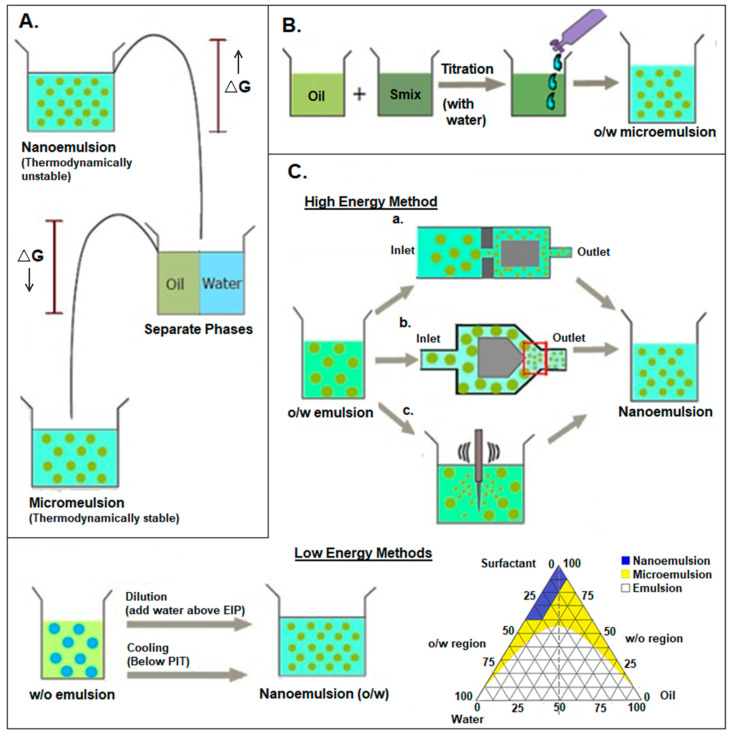
Comparison of nanoemulsions and microemulsions in terms of energy state (**A**); methods of preparation of microemulsions, including high-energy methods, such as piston-gap method (**a**), microfluidization (**b**), and ultrasonication (**c**), and low-energy methods (**B**); and preparation of a microemulsion by the titration method, aided by a pseudoternary phase diagram (**C**). Adapted with permission from [19]. Copyright 2013, Taylor & Francis.

Microemulsions are thermodynamically stable, isotropic emulsions ranging from 100 to 400 nm [11]. The terms microemulsions and nanoemulsions may be a source of confusion as the size of microemulsions may be smaller than the nanoemulsions. This classification system is based on the stability of emulsions and the difference in the preparation process because the microemulsion formed spontaneously, whereas mechanical dispersion should be applied in formulating nanoemulsions [12]. Conventional emulsions are thermodynamically unstable systems, while microemulsions are thermodynamically stable, and nanoemulsions are only kinetically stable and thermodynamically unstable systems (Figure 1A) [13]. The reduction in the oil–water interface and in free energy due to the amphiphilic component makes them more stable, with high loading efficiencies and better contact with biological surfaces for sustained release and enhanced absorption compared to conventional systems [14]. Furthermore, the presence of water and oil phases assists the loading of hydrophilic and hydrophobic compounds in different regions of the emulsified system, rendering an optimal solution for improved solubility and bioavailability [15]. Microemulsions also enable the delivery of therapeutic agents through different routes, including nasal, oral, transdermal, topical, and intravenous, owing to their variable polarity domains that interact through distinct mechanisms with biological systems [16].

#### 2.1.1. Method of Preparation

Compared to the conventional macro- and nanoemulsion, the energy input required for microemulsion is relatively low. The final form is obtained at a low energy state compared to the individual components in the microemulsion system. Therefore, the microemulsion is formed due to a spontaneous process in which energy input is not required (Figure 1B). Although these systems consist of a combination of surfactants and cosurfactants with respective water and oil phases, a high concentration of surfactant is generally enough for spontaneous emulsification [13,17]. Moreover, cosurfactants may be added to form a stable microemulsion. The spontaneous emulsification method, also known as the phase titration method, is used when different proportions of oil and surfactants are titrated against water to construct a phase diagram. A pseudoternary-phase diagram predicts the microemulsion formation zone and consists of three corners, each corner representing 100% of oil, water, or surfactant/cosurfactant mixture (Figure 1C) [18]. The percentage of each component at which a stable emulsion is formed is marked in the diagram, representing the emulsion formation region.

#### 2.1.2. Applications

With distinct polarity domains, spontaneous formulation, and favorable thermal stability, the oil and aqueous phases of a microemulsion can encapsulate both lipophilic and hydrophilic drugs based on the composition and proportion of these phases. Water-in-oil (w/o) microemulsions can be used as a carrier for the delivery of hydrophilic drugs [19], while hydrophobic drugs are efficiently delivered with the help of oil-in-water (o/w) microemulsions [20]. Microemulsions also possess unique characteristics of delivering active pharmaceutical ingredients (APIs) in liquid form, avoiding disintegration and dissolution steps, and the presence of surfactants increases the absorption process, thereby enhancing their bioavailability even when compared to their encapsulation in gelatin shells. 

Another important characteristic of microemulsions is their ability to preserve the drug’s GI stability. Liquid APIs may take 20–30 min to pass through the GI tract compared to solid forms that are trapped for 3 h in the gut, depending on their composition [21]. As solid dosage forms spend more time in the GI tract, they are exposed to enzymatic stress for a longer duration, which may lead to premature degradation. Various studies have demonstrated the encapsulation of poorly water-soluble drugs, such as myricetin, cyclosporin A, ritonavir, and saquinavir, in microemulsions that protect them from oxidative and enzymatic degradation in biological systems [22].

The droplet size of the microemulsion is found in the range of 10 nm to 100 nm or more and possesses a large surface area to volume ratio for absorption of drugs, enhancing their bioavailability. Moreover, a smaller droplet size provides stability due to the higher resistance to sedimentation under the action of gravity, which has been demonstrated to enhance the stability and bioavailability of a wide variety of bioactive molecules such as small molecules such as phenytoin [19], sensitive molecules such as nucleic acids [23], macromolecules such as enzymes [24], peptides [25], and vaccines [26].

In addition to the oral delivery of drugs, topical and transdermal delivery has been extensively investigated using microemulsions. Topical application develops a concentration gradient across the skin through a reservoir effect, leading to drug penetration into the deeper layers of the skin. Consistent diffusion from the external layer provides systemic delivery through the underlying vasculature and protects drugs from hepatic first-pass metabolism. Furthermore, the penetration of poorly permeable drugs can be overcome by adding penetration enhancers such as essential oils [27]. Various studies have improved the therapeutic outcomes of microemulsions by formulating dispersions into gel forms. For instance, transdermal delivery of rutin, acemetacin, curcumin, and rasagiline using microemulsion-based gels has demonstrated the widespread application of this system in various topical and systemic applications [28,29,30].

Microemulsions have found another exciting drug delivery application to the brain over the last few years. Most patients with mental illness suffer from treatment failure due to the impaired transposition of drugs through the blood–brain barrier (BBB) for effective therapy. Intranasal delivery of drugs has shown promising results in bypassing the BBB and delivering drugs directly to the brain. However, intranasal administration of water-insoluble drugs is challenging, and poor absorption from nasal epithelium further compromises brain delivery [31]. Microemulsions have shown promising results in overcoming these limitations by enhancing the solubility and permeability of various drugs and increasing drug delivery to the brain after intranasal administration [32,33]. For instance, brain delivery of zotepine in schizophrenic patients and mebendazole in glioblastoma patients via the intranasal route helps reduce dose-related side effects and improves bioavailability. Its success rate is evident from various FDA-approved microemulsion formulations that target the brain in various disorders [34].

Pickering emulsions are surfactant-free emulsions in which oil droplets are stabilized by a colloidal powder, usually nanoparticles, instead of surfactants. Pickering emulsions have been prepared with droplet sizes as small as 30 nm and can be administered intravenously for systemic applications [35]. Pickering emulsions most appropriately fit the definition of microemulsions as they are thermodynamically stable and are proposed as the most metastable form of emulsions [36]. Recently, the number of articles on synthesis and characterization of Pickering emulsions stabilized by organic polymers or solid particles has increased [37]. To form interfacial barriers, these stabilizing agents should be able to aggregate on droplet surfaces or treated priorly to form colloidal entities, such as nanoparticles, that can be adsorbed on the oil droplets’ surface [38]. For example, a widely investigated flavonoid, quercetin, was transformed into nanocrystals that could adsorb at the oil and water interface to form a Pickering emulsion. The Pickering emulsion resulted in a remarkable increase in bioavailability as compared to pure drugs (2.76 fold) and nanocrystals (1.38 fold) [39].

### 2.2. Nanoemulsions

Nanoemulsions are thermodynamically unstable dispersions of a biphasic system stabilized by a suitable surfactant with a mean diameter of 1 nm to 100 nm. They are blue-white semi-opaque emulsions that might be clear or cloudy in appearance due to their small droplet diameter. This property is occasionally used to differentiate it from a coarse emulsion and microemulsion that usually present a milky white color [40]. These emulsions exhibit unique rheological properties, including viscoelasticity, plasticity, viscosity, droplet size, and structure, based on their chemical composition, phased ratios, nature of surfactants and emulsifiers, and method of preparation. For instance, an increase in the droplet concentration alters the viscosity parameters, whereas a decrease in the droplet size prevents flocculation, sedimentation, and coalescence during the storage and application time frame [41].

#### 2.2.1. Methods of Preparation

Various methods can be used to prepare nanoemulsions (Figure 1C) based on the energy involved, i.e., high-energy and low-energy methods. Emulsifiers with low hydrophilic–lipophilic balance (HLB), between 3 and 6, tend to form w/o emulsions, while those with high HLB of 8–18 form o/w emulsions. Also, the role of ingredients is important in formulating emulsions [42] since a single ingredient can act as a dispersed phase in one emulsion while serving as a continuous phase in another. Mineral oil, as a dispersed phase in o/w emulsions, has an HLB of 11 while acting as a continuous phase with an HLB of 4 in w/o emulsions [43]. High-energy methods include the following: (i) the piston-gap method (Figure 1C(a)), in which a coarse emulsion is passed through a narrow opening under high pressure for size reduction [44,45]; (ii) microfluidization (Figure 1C(b)), in which coarse emulsions is split into channels where they undergo intense movement and high shear mixing in an interaction chamber [46]; and (iii) ultrasonication (Figure 1C(c)), where ultrasonic waves are used to break the larger droplets into smaller ones [47,48]. In low-energy methods (Figure 1C), a w/o type coarse emulsion is converted into an o/w-type nanoemulsion either by lowering the temperature below the phase-inversion temperature (PIT) or by dilution with aqueous phase above the emulsion-inversion point (EIP) [34].

#### 2.2.2. Applications 

Nanoemulsions have smaller droplet sizes, higher kinetic stability, and optical transparency than conventional emulsions. Compared to conventional and microemulsions, nanoemulsions may also present higher physical stability due to their small size and non-Newtonian flow behavior, preventing aggregation and gravitational separation [49,50]. Nanoemulsions are kinetically stable, unaffected by mild changes in pH or temperature. Therefore, nanoemulsions have shown superior protection of encapsulated drugs compared to microemulsions when exposed to higher temperatures and harsh pH conditions [51]. Similarly, their higher tolerance to changes in temperature and pH makes nanoemulsions superior to micro- and macroemulsions in terms of the stability of encapsulated drugs and protection from oxidative, acidic, and enzymatic stress [52]. Nanoemulsions are also employed to encapsulate drugs in oil, thereby protecting them from hydrolytic enzymes, harsh pH, and other environmental stress factors of the GI tract. Therefore, they are excellent candidates for enhancing the bioavailability of water-insoluble drugs by preventing degradation, enhancing solubility, and improving permeability across the GI tract. In addition to high encapsulation efficiencies (>90%), nanoemulsions have also shown the capacity to load drugs above 50% of their total weight (loading capacity > 90%) [53]. Protection of payload from degrading enzymatic environments and acids, when loaded in nanoemulsions, has also attracted interest for the oral delivery of vaccines and peptides, with various studies showing that the protein specificity and integrity are maintained [53,54]. Numerous studies have demonstrated the application of nanoemulsions in various nutraceutical and vitamin formulations to increase their stability and shelf life. Recently, the application of nanoemulsions in photodynamic therapy has opened a new era in the treatment of various skin disorders, where highly hydrophobic photosensitizers have been incorporated in these systems for application at the infected site and irradiated with light of specific wavelengths to increase their therapeutic effects. The encapsulation of methylene blue and the formation of superhydrophobic films and dressings are the most significant advancements in the clinical application of nanoemulsions [55,56].

## 3. Self-Emulsifying Drug Delivery Systems (SEDDS)

SEDDS are classically defined as isotropic mixtures of natural or synthetic oils with either solid or liquid surfactants or a hydrophilic solvent and co-solvents/surfactants system [57]. These mixtures, upon contact with GI fluids and mild agitation provided by the stomach and intestine’s natural movements, result in the formation of fine o/w emulsion. However, recent advances in SEDDS have delineated an extensive interplay between different lipids and surfactants. Hydrophobic ion pairing enables the encapsulation of hydrophilic molecules, particularly biological macromolecules such as nucleic acids, peptides, proteins, and polysaccharides. The pairing of these molecules with auxiliary lipophilic compounds changes the polarity domains and modifies their physicochemical properties to improve therapeutic outcomes [58]. 

### 3.1. Classic SEDDS

Classic SEDDS are stable formulations with advantageous manufacturing ease, while emulsions are sensitive and metastable dispersions. SEDDS are the ideal platform for lipophilic drugs presenting a dissolution rate-limited absorption [59]. These systems improve the rate and extent of absorption, with blood time profiles being reproduced more easily. These systems produce droplets of very small size during emulsification inside the biological system, which improves transportation and absorption through different pathways, including the lymphatic system. Rationally, SEDDS can be used for several classes of drugs, as outlined in the biopharmaceutical classification system (BCS), with varying solubility or permeability, which may limit their absorption [60]. 

SEDDS are isotropic mixtures of oil, surfactant, and co-surfactant/co-solvent with a suitable API. Their selection was based on various factors, including drug solubility and miscibility, solubilization capacity of the oils and surfactant, self-emulsification abilities, toxicities, purity, and chemical compatibility of all the components. The concentration ratios of these components are critical for developing optimized SEDDS. Oil is the first major ingredient of SEDDS formulations because it not only solubilizes various lipophilic drugs as a matrix of SEDDS but also enhances absorption of the drug from the GI tract via the intestinal lymphatic system [61]. Triglyceride oils with different chain lengths, such as medium- and long-chain triglycerides, having varying degrees of saturation, are commonly used in SEDDS synthesis. Medium-chain triglycerides have 6–12 carbon atoms and are directly transported to the systemic circulation via portal blood. At the same time, long-chain triglycerides, with more than 12 carbon atoms, are transported via the intestinal lymphatic system. Medium-chain triglycerides have attained the upper edge over long-chain triglycerides because of their high solvent capacity and oxidation resistance, so they are preferred in lipid-based formulations [62]. Edible oils, due to their low solubility profile for lipophilic drugs, have not been used to develop SEDDS. Specific lipid components also trigger the natural synthesis and release of digestive secretions from the pancreas and liver in the form of pancreatic juice and bile salts, which play a significant role in the emulsification process and improve the solubility of the API [63].

Upon hydration, surfactants are preferentially adsorbed at the oil–water interface and facilitate oil dispersion as fine droplets in gastric media, facilitating the solubility of larger quantities of drugs without precipitation because of their amphiphilic nature [64]. Different surfactants can be used to formulate SEDDS, but non-ionic surfactants with a high HLB are more frequently used. Natural emulsifiers, such as lecithin and fatty acid-derived surfactants, are preferred over synthetic emulsifiers because of their safety and cost-effectiveness. However, their inadequate self-emulsification capacity limits their use on a larger scale. Non-ionic surfactants are less toxic and stable at a broader spectrum of GI pH and ionic concentrations than ionic surfactants, which are less toxic but might reversibly alter intestinal lumen permeability [65]. Generally, stable SEDDS are formed by surfactant concentrations ranging from 30 to 60% *w*/*w*. Furthermore, it is also important to measure optimum surfactant concentration as a large amount of surfactant might lead to GI irritation or other health risks. Co-surfactants, also termed co-solvents, are added to achieve a stable and effective formulation by guaranteeing the flexibility of the interfacial layer as it decreases the interfacial tension, therefore acquiring different curvatures as required to form microemulsions over an extensive composition range [66]. Alcohols with carbon atoms ranging from 3 to 8 (medium-chain alcohols) are frequently used as co-surfactants. Ethyl alcohol, propylene glycol, and polyethylene glycol dissolve large quantities of hydrophilic surfactants [64]. The lipid mixture with high surfactant and co-surfactant/oil ratios will result in SEDDS formulations. 

The storage process of emulsions can be a challenge, as their instability may lead to undesirable changes in the formulation over time. In contrast, SEDDS are thermodynamically stable and can be easily stored for longer periods [67]. As SEDDS have no aqueous content, the phase separation problems are avoided compared to microemulsions or nanoemulsions. Solid dosage forms are preferred due to ease of handling and prolonged stability [68]. Keeping this in mind, solid SEDDS formulations were prepared by spraying SEDDS on them and molding them into tablets, as they are easy to administer and store, which may also enhance patient compliance [69]. Palatability is a major concern for lipid-based formulations, which can be solved by filling SEDDS into capsules [70]. Formulations of drugs such as SEDDS also prevent undesired effects related to food intake, such as changes in the pH through the GI environment. SEDDS are more feasible for industrial scale-up as they involve simple and cost-effective facilities such as agitator mixers and liquid filling machines [71]. 

### 3.2. Self-Micro Emulsifying Drug Delivery System (SMEDDS)

A self-microemulsifying drug delivery system (SMEDDS) refers to a formulation that produces transparent microemulsions with oil droplets upon hydration. The resulting droplets range in size from 10 to 500 nm. SMEDDS are easy-to-prepare members of the self-emulsifying family where, firstly, the drug is dissolved in a specific amount of oil, or a mixture of oils, and then the surfactant and co-surfactants are added and mixed to obtain a clear, transparent formulation [72]. However, the SMEDDS has its own intricate variables associated with its stability and scalability. These systems have the advantage of higher loading efficiency with minimal effect on food in the GI tract. Variability in the absorption of various therapeutic agents owing to the presence or absence of food can be modified by formulating them in SMEDDS. For instance, dronedarone, an antiarrhythmic drug, has a 10-fold difference in absorbance in the fasting and postprandial phases, which can be reduced 2 fold by formulation in the form of an SMEDDS, thereby improving clinical efficacy and patient compliance [73]. 

Bisdemethoxycurcumin is a novel nutraceutical agent recognized for its antimutagenic properties, but its application is limited by its low solubility. Bisdemethoxycurcumin SMEDDS were prepared using ethyl oleate as the oil counterpart, Kolliphor EL as a surfactant, and polyethylene glycol (PEG) 400 as a co-surfactant. The SMEDDS enhanced the nutraceutical agent bioavailability by 3.7 times and was independent of pH variation of the GI tract [74]. Carvedilol is a low-solubility drug with incomplete release in vivo, leading to low bioavailability. Formulation of carvedilol as SMEDDS led to complete drug release within 10 min, whereas the release of pure carvedilol remained incomplete after several hours [75]. In another example, CAT3, an antitumor agent that can treat glioblastoma, was loaded into SMEEDS to avert early in vivo metabolism to the active metabolite PF403, which causes severe GI side effects and enhances its permeability by 3.9 times. SMEDDS was also shown to promote lymphatic transport, leading to increased bioavailability of CAT3 (79%) and PF403 (49%) [76]. SMEDDS has recently been assessed for intramuscular injection of drugs being considered safe and able to increase the solubility of the water-insoluble drug diclofenac [77]. Similarly, the SEDDS formulation of desmopressin demonstrated projective effects by preserving thiol–disulfide exchange reactions [78]. Mucosal permeability significantly affects the systemic absorption and bioavailability of various APIs. Numerous studies have reported that SMEDDS have better mucosal permeability than other nanocarriers, such as liposomes and solid lipid nanoparticles. Generally, smaller droplet sizes and negative zeta potential are considered to promote mucosal permeation [79].

### 3.3. Self-Nano-Emulsifying Drug Delivery Systems (SNEDDS)

Self-nano-emulsifying drug delivery systems (SNEDDS) yield nanoemulsion-like droplets with a size of less than 100 nm. The preparation of SNEDDS is like that of SMEDDS, in which the drug, in the appropriate amount, is solubilized in the oil, and surfactant and co-surfactant are added to it. This mixture is vortexed until an isotropic mixture is obtained [77]. Spontaneous emulsification occurs when the entropy change exceeds the energy required for surface area expansion. Compared to the nanostructured lipid carriers, liposomes, noisomes, and solid lipid nanoparticles, SNEDDS did not face the traditional challenges of particle aggregation and sedimentation, as they only produced spontaneously inside the GI. 

SNEDDS are also extensively used to enhance the solubility and bioavailability of water-insoluble drugs. For example, clopidogrel-loaded SNEDDS, coated on Aeroperl 300, led to a 9-times increase in its bioavailability [80]. β-cyclodextrin is widely used to form solid dispersions of water-insoluble drugs to enhance their solubility. SNEDDS were combined with β-cyclodextrin to form innovative solid SNEDDS that enhanced the solubility and bioavailability (AUC) of dexibuprofen to a greater extent than the free drug or a solid dispersion alone. Compared to other solid SNEDDS, β-cyclodextrin-based solid SNEDDS can offer superior flow properties due to their spherical shape [81]. In another study, the inclusion of hydroxypropyl β-cyclodextrin, a hydrophobic derivative of β-cyclodextrin, enabled the design of solid-state SNEDDS and provided around 5 and 1.4 times higher Cmax and 2 and 1.7 times higher bioavailability than pure drug or standard solid-dispersions, respectively [82]. Numerous studies have demonstrated the different formulation parameters that influence the therapeutic performance of SNEDDS, with a prime focus on the delivery of various therapeutic agents for the treatment of diabetes, pancreatic cancer, neurodegenerative diseases, and immune diseases.

The application of lipid-based drug delivery systems has often been challenged for metabolic disorders, especially diseases that involve altered lipid profiles. To address this issue, we developed fish-oil-based SNEDDS for the delivery of the cardioprotective drug rosuvastatin. Fish oils are rich in omega-3 fatty acids and have the potential to synergize the therapeutic activity of rosuvastatin. These SNEDDS offer excellent solubility enhancement, drug release, stability, and emulsification profile, which makes them an ideal carrier for lipophilic drugs [83]. Later, we used flaxseed oil as a rich source of omega-3 fatty acids to develop SNEDDS of curcumin, a well-known bioactive compound. The synergistic anti-inflammatory action was demonstrated by 2 and 3 times higher percentage inhibition than curcumin and flaxseed oil alone, respectively, in the carrageenan-induced paw inflammation model [84]. SNEDDS of bioactive oils has been reported to potentiate the therapeutic effects of loaded drugs. For example, blackseed oil is reported for gastroprotective properties, and its incorporation in curcumin and piperine-loaded SNEDDS was found to provide adequate biocompatibility and synergistic cytotoxicity to several types of cancer cells in the absence of chemotherapeutic drugs [85,86]. 

Nearly 12 self-emulsifying drug delivery systems based on SEDDS, SMEDDS, or SNEDDS formulations are available in the market, and many more are in clinical trials [87]. Therefore, self-emulsifying drug delivery systems have an enormous potential to enhance the solubility and bioavailability of water-insoluble drugs, and their combination with solid dispersions or the use of bioactive oils further expands their applications in the field of drug delivery.

## 4. Solid-Phase Lipid Nanoparticles: Solid Lipid Nanoparticles and Nanostructured Lipid Nanoparticles

Lipid nanoparticles are prepared from a solid-state lipid matrix and are termed SLNs. Their structure resembles one of nanoemulsions, except that the SLNs contain a solid lipid matrix coated with a surfactant layer [88,89]. Compared to nanoparticles made from hydrophobic polymers, SLNs consist of natural and synthetic lipids with building blocks of fatty acids. SLNs offer unique advantages due to their solid nature, being the most relevant, the controlled release of encapsulated drugs over a long time due to the presence of solid lipids that resist the penetration of water to the SLN core and the lack of mobility of drug molecules in the lipid matrix [90]. SLNs are mainly used to deliver water-insoluble drugs that can be mixed directly with the lipid phase during preparation. Generally, the microemulsion method is used for the preparation of lipid nanoparticles. First, the lipid is melted to an oil-like liquid phase above its melting point. Then, the lipid phase is emulsified with an aqueous surfactant solution and heated at a high temperature. This hot microemulsion is then cooled down to form SLNs. Otherwise, the hot microemulsion can be added to the cold aqueous phase (4 °C) to form SLNs [91]. The microemulsion method spares the drugs from potentially toxic organic solvents required for synthesizing polymeric or other lipid-based nanoparticles [92,93]. Furthermore, lipid nanoparticles are usually made from natural lipids that make them biocompatible [94,95]. 

**Figure 2:**
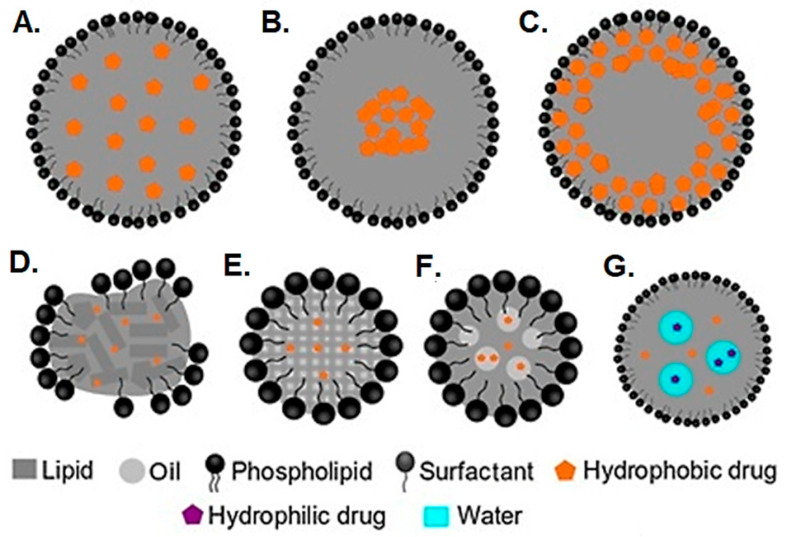
Structure and distribution of drugs in solid-phase lipid nanoparticles: uniform distribution of a drug inside SLNs (**A**), drug-enriched core of SLNs (**B**), drug concentrated in the shell of the SLNs (**C**), lipid crystal formation in SLNs leading to a disc-shaped unstable state (**D**), NLC structure with homogenous lipid phase of oil and lipid (**E**), presence of oil globule inside NLCs due to a higher amount of drug oil (**F**), and multicompartment SLNs with aqueous cores (**G**). Reproduced with modifications (CC BY 4.0) from [97].

Initially, drugs and lipids were believed to compete for the same place in the lipid nanoparticles. If the lipid concentration is low (<10%), it will remain embedded inside drug-enriched shells (Figure 2A). However, the lipid can form a protective shell over the drug when the lipid concentration is higher than 20% [96]. Later, it was found that the method of preparation of lipid nanoparticles plays a more pivotal role in forming a core–shell structure. In general, if the drug precipitates before the lipid in the cooling step, the drug is localized in the core of the SLNs (Figure 2B). On the other hand, faster cooling of a hot microemulsion can lead to precipitation of lipids before the drug and localization of the drug in the shell of the SLNs (Figure 2C). The drug in the shell will be released more rapidly than the drug in the core of the SLNs [97]. Therefore, the selection of lipid-to-drug ratio and the pattern of cooling needs to be fine-tuned to control the different characteristics of nanoparticles obtained. Many lipids used to manufacture SLNs are present in crystalline form due to the orientation of the carbon chain. In the cooling step of the preparation of SLNS, the carbon chains of lipids are placed haphazardly and start to resume their orientation with time, leading to the growth of lipid crystals [88]. This causes round-shaped SLNs to transform into a disc-like shape and exposes new surfaces that are not coated with surfactant (Figure 2D). SLNs start to aggregate with each other due to sticky, hydrophobic surfaces, leading to the formation of a gel-like phase. However, adding a small amount of oil to solid lipids makes a heterogeneous lipid mixture with a low tendency to crystallize [98]. 

NLCs are a modification of SLNs in which a liquid lipid or oil is added to the solid lipid before the preparation of the nanoparticle. Due to the addition of oil, NLCs have higher stability and loading efficiency [99]. A combination of oil and solid lipids offers a heterogenous lipid mixture to encapsulate the drug and avoid crystallization (Figure 2E). If the oil concentration is too high, patches of oil can form (Figure 2F). Both SLNs and NLCs are prepared using similar methods. Almost always, the product quality can be further improved by sonication, homogenization, and extrusion to reduce particle size and ensure homogenous size distribution [100]. In addition, the preparation method of SLNs and NLCs is readily scalable for industrial production, and they are already being marketed for cosmetic applications [101,102,103]. SLNs can also be made to encapsulate hydrophilic drugs by suitable selection of the lipid phase, surfactant system, or development of the multicompartment lipid nanoparticles with an inner aqueous phase. Hence, SLNs and NLCs may be prepared to load simultaneously water-soluble and insoluble drugs (Figure 2G), a property previously assigned to liposomes [91]. The encapsulation efficiency of drugs is higher in NLCs than in SLNs due to the incorporation of oil into the solid lipids. Several studies have been performed to screen oils against drugs and select the oil with the highest drug solubility. The incorporation of the highest solubility oil is responsible for the higher encapsulation of NLCs [104]. Initially termed simply as binary SLNs, NLCs represent the second generation of lipid nanoparticles and are thought to replace the SLNs due to the similar structure but added advantages of higher encapsulation efficiency and improved colloidal stability. 

### 4.1. Applications

The SLNs are employed in all general applications of lipid nanoparticles where existence in a solid state offers additional benefits. Their salient applications are discussed below. 

#### 4.1.1. Controlled Release of Drugs 

Typically, nanoemulsions release the drug immediately depending upon the drug’s partition coefficient in the oil phase. However, SLNs and NLCs resist the penetration of water as well as the diffusion of drugs due to their solid nature. As the rate of drug release from the SLNs also depends upon diffusion path length, it is pertinent to discuss the presence of a drug in either the core or shell of the SLNs. Therefore, the SLNs and NLCs have been used to deliver all types of drugs and diagnostic agents.

Regarding solubility, hydrophilic drugs are released more rapidly and show lower encapsulation efficiency than hydrophobic drugs. 5-Fluorouracil (5-FU) is a water-soluble drug (1 mg/mL) used to treat various cancers. However, it has low miscibility with lipids and shows low encapsulation efficiency in SLNs (around 40%) [105]. This challenge is overcome by designing a surfactant shell around SLNs enriched with the drug. For example, when lecithin and poloxamer 188 are used as surfactant systems, they form reverse micelles within the SLNs to encapsulate a higher amount of 5-FU, and an encapsulation efficiency of up to 50–60% can be achieved [106]. Like multiple emulsions, the multicompartment SLNs can be prepared with a water-in-oil-in-water structure wherein hydrophilic drugs can be incorporated into the inner aqueous core (Figure 2G). Indeed, the multicompartment SLNs have been used to load 5-FU in the inner aqueous core with an average entrapment efficiency (EE) of 74% [107]. We have employed multicompartment SLNs for the safe delivery of insulin, which remains a key challenge in pharmaceutical sciences. After oral administration to rats, SLNs provided a significant hypoglycemic effect for 24 h. Interestingly, coating SLNs with chitosan improves their mucoadhesion in GIT and increases bioavailability by more than 2 times [108,109]. SLNs can be lyophilized to a fine powder and stored for years. The insulin-loaded SLNs can be lyophilized with or without cryoprotectant while maintaining the secondary structure and biological function of insulin (84%) under accelerated stability conditions (40 °C/75% RH) [110]. However, SLNs cannot claim the status of “ideal” peptide carriers due to the promising performance of other nanocarriers, such as porous silicon and polymeric (PLGA) nanoparticles [111]. Therefore, multiple-type SLNs offer a unique platform for enhanced encapsulation and controlled release of water-soluble drugs in vitro and in vivo. The additional benefit of multicompartment SLNs is their improved stability as compared to multiple emulsions that are thermodynamically unstable and have an increased risk of flocculation, coalescence, and creaming [112]. Also, breaking multiple emulsions would result in a simple emulsion and loss of its function. However, solid lipid matrices in SLNs or NLCs lack these problems.

#### 4.1.2. Intravenous Delivery of Drugs

The first application of the SLNs was their suitability for intravenous (IV) injection. Emulsions show a rapid release of drugs, and their IV applications are minimal [113]. However, SLNs present themselves as an ideal lipid-based DDS for controlled and targeted delivery applications. SLNs show prolonged blood circulations as the hydrophilic groups of the surfactants impart stealth properties. After injection, the proteins present in the blood may interact with SLNs and get adsorbed at their surface, posing a different challenge than the one traditionally found for protein interactions with drug molecules. Of foremost importance are the opsonins, the proteins of the immune system that become adsorbed on the SLN surface and facilitate their recognition and removal by the phagocytic cells of the immune system. On the other hand, some proteins, such as albumin and apolipoproteins, may prevent phagocytic cell uptake after adsorption on the surface of SLNs. These proteins are termed dysopsonins and prolong the circulation time of the SLNs [114]. By taking advantage of this feature, SLNs have been assessed for administering dexamethasone to the lungs by inhalation. In vitro studies showed that the dexamethasone release was sustained for 48 h, which is superior to currently available treatment options. In vivo studies on mice showed that around 18 times higher level of drug was achieved in the lungs, compared to the drug solution, within 0.5 h of injection [115]. Cationic SLNs, consisting of cationic lipids or cationic surfactants, can bind the negatively charged siRNA on their surface while the anticancer drugs are encapsulated in the core. This combinatorial approach provides silencing of target genes and chemotherapeutic cytotoxicity to kill the drug-resistant cancer cells [116].

The SLNs can be made to target specific tissues by attachment of a targeting ligand. For example, folic acid-conjugated and paclitaxel-loaded SLNs have shown higher cytotoxicity, i.e., 10 and 3 times lower IC50 values than pure drug and unconjugated SLB, respectively, for human lung carcinoma cells (A549). Folic acid coating also leads to 4 times higher uptake by the cancer cells as compared to unconjugated nanoparticles with different lipid compositions [117]. These results were potentiated by the enhanced cellular uptake mediated by the folate receptor of folic acid-modified SLNs. Folic acid-conjugated chitosan is widely used to form a stable ligand coated over SLNs. This strategy was successfully employed to improve the bioactivity of Artemisia vulgaris essential oil and achieve significant antimicrobial and anticancer activity as compared to the pure drug [118]. More recently, SLNs have been used to reverse cancer resistance. The intrinsic ability of the SLNs to cross the cell membrane and penetrate inside the cell along with encapsulated drugs prevents drug efflux by p-glycoproteins (p-gp) pumps, a leading mechanism of cancer resistance [119]. 

#### 4.1.3. Targeted Delivery to the Brain

**Figure 3:**
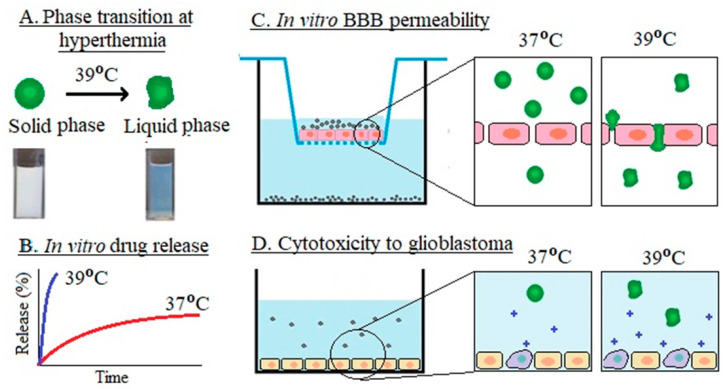
Application of thermoresponsive lipid nanoparticles targeting cancer. Lipid nanoparticles melt under hyperthermia (**A**), abrupt release of 5-FU under hyperthermia (**B**), nanoparticles under hyperthermia can squeeze through the BBB (**C**), and higher cytotoxicity to cancer cells under hyperthermia (**D**). Reproduced with modifications (CC BY 4.0) from [125].

Due to their small size and lipophilic nature, most current SLN research focuses on targeted delivery to the brain due to the high passive targeting of lipid nanoparticles across the BBB, making them ideal candidates to deliver payload across the BBB [120]. Apolipoprotein E (Apo E) is a protein taken up by the brain by ApoE receptors present in the BBB. ApoE-functionalized SLNs can benefit from this receptor-mediated transport and deliver the payload across the BBB when administered through inhalation [121] or IV routes [122]. Another factor leading to the low efficacy of chemotherapy is the presence of p-glycoprotein (p-gp) efflux pumps in endothelial cells of the BBB as well as multidrug-resistant glioblastoma cells. It was shown that surfactants used to prepare SLNs, such as Brij 78, can inhibit the p-gp efflux pump from increasing the localization of drugs at the target site [123]. In addition, p-gp efflux pump inhibitors are co-loaded in SLNs to inhibit the removal of the drugs and increase their localization in the brain. Recently, we have developed first-of-its-kind lipid nanoparticles that remain solid at normal body temperature but undergo a solid–liquid-phase transition at hyperthermia, resulting in liquid-phase nanoparticles [124]. These nanoparticles were termed thermoresponsive lipid nanoparticles (TLN) due to their existence in both the solid and the liquid state, like nanoemulsions. The TLN showed minimum drug release for 5-fluorouracil (5-FU), i.e., 50–80% drug release in 60 h at normal body temperature, which is characteristic of the SLNs, whereas they melt to a liquid state at hyperthermia (39 °C), leading to faster drug release within 4–5 h. We have also demonstrated that the TLN showed higher penetration across the in vitro BBB model at hyperthermia (Figure 3). This is due to the liquid state at hyperthermia, as demonstrated previously for nanoemulsions [125]. More recently, we have prepared TLN from a eutectic mixture of lipids in which the thermoresponsive lipids presented a melting point (41 °C) lower than the fatty acid components, such as stearic acid (69 °C), palmitic acid (62.9 °C), and myristic acid (54 °C). The nanoparticles showed excellent biocompatibility, significantly higher cytotoxicity under hyperthermia (41 °C) as compared to normothermia (37 °C), and 23 times higher uptake by MCF-7 breast cancer cells as compared to pure drug solution [unpublished].

In addition to cancer, SLNs and NLCs have been extensively studied after other neurodegenerative diseases. RVG29 peptide is the most widely used targeting ligand aimed at the neurons. Treatment of Alzheimer’s disease is a prime example wherever BBB restricts lipid nanoparticle entry into the brain. Lipid nanoparticles functionalized with RVG29 peptide have shown 1.5-fold higher permeability across BBB within 4 h to deliver quercetin, a proven agent for treating Alzheimer’s disease [126]. 

Nanoparticles possess high surface energy, which leads to surface adsorption of biomolecules in blood, mainly proteins, to form a protein corona (PC). PC on lipid-based nanoparticles is not widely investigated because they resist the adsorption of proteins due to the presence of hydrophilic or polar head groups on surfactant-coated nanoparticles or occasional coating with hydrophilic polymers, such as PEG. Yet, researchers have utilized PC to regulate the biological fate of nanoparticles, such as SLN and NLC. For example, the surfactant’s hydrophilic chain length can be tuned to promote the binding of apolipoprotein J (ApoJ), which reduces the interaction with immune components and phagocytic cells [127]. This is an interesting strategy to prolong the blood circulation of nanoparticles without altering their structure or attachment of ligands. Taking a step forward, the attachment of apolipoprotein E (ApoE) to NLC has been shown to cross the BBB by utilizing receptor-mediated transcytosis of ApoE. ApoE can be physically adsorbed by modifying nanoparticles to a discoid shape [128] or attached by covalent linkers [129]. 

#### 4.1.4. Topical Delivery of Drugs and Cosmetics

Like emulsions, the SLNs and the NLCs were widely employed for smoothening the skin. Lipid nanoparticles are adsorbed in the thin lipid film stratum corneum, re-establishing its integrity and being essential to maintaining skin hydration. The lipid nanoparticles loaded with drugs or cosmetic agents act as a reservoir, releasing the payload to the skin at a predetermined rate. SLNs were compared to liposomes, ointments, creams, and hydrophilic gels to deliver benzyl nicotine to the skin, showing superior efficacy in terms of fast delivery, the maximum amount delivered, and sustained drug release [130]. In a comparative study, SLNs, NLCs, and liposomes exhibited 11.5, 12.5, and 3.7 times enhanced bioavailability of tacrolimus, respectively, after topical application as compared to pure tacrolimus solution [131]. NLCs were combined with antimicrobial silver for the treatment of atopic dermatitis, offering a barrier function to inhibit skin water loss and prevent systemic absorption of the silver and, thus, prevent silver-related side effects [132]. Up till now, most solid-phase lipid nanoparticles for topical delivery are designed for cosmetic agents. For example, SLNs and NLCs were prepared to deliver resveratrol, vitamin E, and epigallocatechin gallate. Both lipid nanoparticles showed sustained release for 24 h and enhanced absorption through the skin [133]. However, a better understanding of the interaction of lipid nanoparticles with skin components is necessary for these combinatorial delivery applications. 

## 5. Vesicular Drug Delivery Systems: Liposomes and Niosomes

Vesicles have emerged as pharmaceutical carriers of choice in recent decades due to their ability to enhance the bioavailability of water-soluble and insoluble drugs [134]. Compared to matrix-type solid- and liquid-phase nanoparticles described previously, the vesicles have a well-defined aqueous core surrounded by a bilayer of lipids or lipid derivatives.

Liposomes, first studied by A.D. Bangham in 1965, were developed with different phospholipids and investigated extensively as drug carrier modules. Liposomes have been widely used as pharmaceutical vehicles owing to key attributes such as accommodating lipophobic or lipophilic drugs, protection of encapsulated moieties from undesired effects of the external environment, functionalization with specific ligands, and coating with biocompatible and inert polymers [135]. The presence of natural lipids also brings some drawbacks, such as instability, difficulty in heat sterilization, and complex scale-up.

Niosomes are a new version of vesicles, developed from non-ionic surfactants, that can be developed by self-assembling non-ionic amphiphiles in bilayered or multilayered vesicles, with or without cholesterol as an additive. Such vesicles can exhibit remarkable advantages compared to conventional lipid vesicles (e.g., liposomes), such as larger formulation versatility, more options for sterilization, extended shelf-life, and improved stability profile [135]. Small drug molecules, macromolecules (proteins), nucleotides, and plasmids (bacterial DNA) have been incorporated into liposomes and niosomes for evaluation of their delivery [136]. 

### 5.1. Formulation and Preparation Methods

Liposomes consist specifically of phospholipids, requiring specific ingredients to stabilize their lipid bilayer, while niosomes consist of non-ionic surfactants. Therefore, the design of a suitable drug delivery system requires basic information regarding its different components since they can affect the product’s performance during manufacture as well as during application. Therefore, a brief note on vesicular systems’ formulation components is provided.

Phospholipids, from different classes, are the major constituents in the formation of liposomes. Many formulations use synthetic derivatives of natural phospholipids, mainly phosphatidylcholine and derivatives, including distearoyl phosphatidylserine (DSPS), dilauryl phosphatidylglycerol (DLPG), dioleoyl phosphatidylethanolamine (DOPE), dilauryl phosphatidylcholine (DLPC), dimyristoyl phosphatidylethanolamine (DMPE), dilauryl phosphatidylethanolamine (DLPE), dioleolyl phosphatidylcholine (DOPC), dimyristoyl phosphatidylcholine (DMPC), dipalmitoyl phosphatidylcholine (DPPC), and distearoyl phosphatidylcholine (DSPC) [137]. 

Non-ionic amphiphiles contain well–defined regions with varying solubility, i.e., a lipophilic (organic-soluble) and a hydrophilic (water-soluble) end [138]. Major classes of non-ionic surfactants used for niosomes preparation include polyoxyethylene sorbitan fatty acid esters (Tweens), sorbitan fatty acid esters (Spans), alkyl ethers (Brij), and alkyl glyceryl ether [139]. New surfactants used for the preparation of niosomes include Gemini surfactants with more than one hydrophilic head group, Bola amphiphiles with two hydrophilic groups attached by a long-chain spacer [140], and Pluronic block copolymers [141] that offer unique properties in terms of tunable hydrophilicity, low CMC, and unique vesicular structures.

When creating vesicles in both liposomes and niosomes, cholesterol is a crucial component that provides stability to the structure. It helps to increase cohesion among the carbon chain and rigidity of the membrane, preventing any leakage of the payload [142]. It is worth mentioning that minute quantities of charged lipids are mostly added to stabilize vesicle dispersion against aggregation. As niosomes consist of non-ionic surfactants, charge-inducing agents are generally added to provide sufficient steric stabilization, such as positive charge inducers (e.g., stearyl amine and cetyl pyridinium chloride) or negative charge inducers (e.g., diacetyl phosphate and phosphatidic acid) [143]. Generally, a charged molecule is added at a percent mole ratio of 2.5–5% because higher concentrations can probably inhibit the formation of niosomes [144].

Niosomes and liposomes are prepared using the thin film hydration method, which involves depositing a lipid layer in a round bottom flask by vaporizing its organic solvents, generally chloroform or ethanol. The thin film is then hydrated with phosphate-buffered saline (PBS) to obtain the vesicules [143]. The resulting vesicles are primarily large multilamellar structures possessing multiple lipid bilayers. An extrusion step is typically employed to create smaller and more uniform unilamellar vesicles. This method is simple and can be performed without the need for special equipment. Alternatively, the ether injection method can be used for higher drug loading, which involves the injection of a lipid phase in diethyl ether into an aqueous phase above the phase transition temperature. Then, evaporation of ether by rotary evaporation results in the development of single-layered vesicles [145]. Liposome technology has also benefitted from advancements in microfluidics. Smaller-sized vesicles with higher uniformity and unilamellar configuration are achieved by utilizing the microfluidization approach. This method is based on a submerged-jet principle, which involves the interaction of two fluidized streams at terminal velocities in precisely designed micro paths. Vesicle formation occurs in a region of maximum energy, which is attained owing to the impingement of a thin liquid sheet along a common front [145]. The ultrasonic processing (UP) technique is the latest development in this field and uses the energy of ultrasound waves to form vesicles. The ultrasound waves are generated by a probe dipped in a mixture of lipid or surfactant, cholesterol, and a charge-inducing agent in water [146]. The effect of amplitude and sonication time on vesical size, polydispersity index (PDI), and entrapment efficiency needs to be optimized to produce niosomes by this technique. In a study, a high-intensity probe sonicator (power: 750 W; frequency: 20 kHz) was used at three levels of amplitude (50%, 70%, and 90%) and sonication time (15, 30, and 45 min) in pulsed mode (50 s pulses and 10 s pause) at a probe temperature of 57 °C. The aim was to assess the optimal experimental conditions for producing niosomes. The most homogeneous formulation, with a PDI of 0.27, a particle size of 405 nm, and a high EE of 75.1%, was achieved using 70% ultrasound amplitude and 45 min of sonication time. The overall results revealed the potency of the UP technique as a fast, economical, and green preparation protocol for developing niosomes [147]. 

### 5.2. Applications 

#### 5.2.1. Gene Delivery

Nucleic acid (RNA and DNA)-based drugs carry negative charges, and liposomes have shown promise in their delivery due to their ability to encapsulate and protect them inside an aqueous core. Many positively charged lipids have been incorporated in liposomes (termed lipoplexes) and niosomes to form ion pairs with nucleic acids to increase payload and prevent leakage. Cationic lipids can be prepared as cholesterol derivatives or alkyl lipids carrying positively charged groups, although positively charged lipids are more widely used [148].

Liposomes containing lipoconjugates of different PEG chain lengths were evaluated in mice to demonstrate their role in siRNA-mediated immunotherapy. Coatings with long PEG chains induced the synthesis of monocyte chemoattractant protein-1 (MCP-1) and interferons (INF-α, β, and γ), and short PEG chain coatings activated only MCP-1 and INF-γ in mice, depicting a remarkable difference in their interaction with different immune components. Surprisingly, uncoated liposomes with siRNA pre-complexes provided the highest tumoricidal activity in vitro and in vivo, whereas liposomes with long PEG chains produced negligible action since the PEG chains hindered the interaction of liposomes with cell membranes to a level where they could not enter the cell and resulted in the loss of therapeutic activity [149].

Niosomes prepared with cationic lipid 2,3-di(tetradecyloxy)propane-1-amine and Tween 80 have been used for gene delivery. Mesenchymal stem cells (MSCs) transfected with niosomal vesicles demonstrated increased growth rate, improved alkaline phosphatase activity (ALP), and extracellular matrix deposition, which suggested the formation of osteoblast-like cells leading to bone regeneration [150]. The critical role of cholesterol in promoting RNA transfection in the body is widely demonstrated. Niosomes containing a surfactant mixture, cholesterol, and a cationic lipid in the 1:1:0.25 ratio showed the highest gene transfection efficiency [151]. The cholesterol/lipid ratio (1:1) required for efficient gene delivery is higher than that required for small molecular drugs (7:6–2:1) [152]. Cationic niosomes, prepared with cationic lipids with a dimethylamino head group, achieved higher transfection capacity when compared with their neutral counterparts [153]. These niosomes also exhibited higher cytotoxicity, especially in ARPE-19 cells, at cationic lipid/DNA ratios of 20:1 and 30:1 [153].

The toxicity of cationic liposomes was attributed to the non-specific interaction with negatively charged body cells. However, detailed mechanistic studies revealed that the production of reactive oxygen species (ROS) and alteration of cellular metabolism and key signaling pathways may also be involved [154]. Therefore, comprehensive characterization of cationic liposomes and niosomes must be carried out on a case-by-case basis to establish their safety profile.

#### 5.2.2. Vaccine Delivery

The demand for faster and more successful vaccine production systems is a challenge in public health. Recently, nucleic acid-based vaccines emerged as a promising approach for the development of safe and effective vaccines against a wide range of pathologies, including infectious diseases, cancer, and other non-infectious diseases with a high socioeconomic impact in modern societies. While traditional vaccines use live or inactivated pathogens or subunit antigens, nucleic acid-based vaccines comprise the delivery of nucleic acid (plasmid DNA or messenger RNA—mRNA) encoding the antigen of interest for a certain disease. Upon intracellular delivery, the nucleic acids are processed by the cell mechanisms, leading to the translation into protein antigens that enable an immune response, which is responsible for producing specific antibodies and activation of T cells that protect against the target pathogen or disease [155,156,157,158,159]. 

Several advantages can be attributed to nucleic acid-based vaccines compared to their traditional counterparts. First, they can induce both humoral and cellular immune responses. Furthermore, they are cost-effective in their industrial production and can be quickly adapted to provide broad-spectrum protection against multiple strains or variants of a pathogen [160]. Nevertheless, there are still some challenges for nucleic acid-based vaccines. The major problem concerns their delivery to target cells in vivo, as naked nucleic acids are rapidly degraded by nucleases and present poor uptake and expression efficiency in vivo [161]. Therefore, developing efficient delivery systems is pivotal for the success of nucleic acid-based vaccines and their implementation as a therapeutic standard. An ideal delivery system for nucleic acid-based vaccines should ensure the protection of the nucleic acids from degradation, simultaneously promote cellular uptake, and enable intracellular release and expression.

Liposomes offer additional advantages of acting as immune adjuncts, ameliorating humoral and cell-mediated immune responses [162]. The effects of epitope density and physicochemical aspects of liposomes, such as membrane fluidity and charge density, have been widely investigated [163]. The Swiss Serum and Vaccine Institute, Berne, Switzerland, has recently developed a liposome-based hepatitis A vaccine (Epaxal1) and tested it in humans. Influenza virus hemagglutinin and inactivated hepatitis A virus particles have been incorporated into this vaccine [164]. Moreover, vesicles have also been used to treat various diseases on the molecular level through gene therapy. Gene therapy involves the modification or insertion of a new gene. Liposomes encapsulating nucleic acids can transfect certain cells upon local or systemic administration. It was noticed that immunization via the topical route produces memory responses like those produced by the oral route. Niosomes presented a higher degree of immune response than liposomes. Transdermal delivery of niosomes against tetanus toxoid was comparable to outcomes achieved with intramuscular injection of an equivalent dose of antitoxoid tetanus [165,166].

#### 5.2.3. Anticancer Drug Delivery

Based on a net surface charge, three classes of liposomes have been utilized for anticancer drug delivery. Neutral, positive, and negatively charged liposomes are mostly employed for this purpose. Lymph nodes provide a site for the accumulation of liposomes, which leads to enhanced delivery of drugs to rapidly growing cancer cells or reduces the virus load of HIV-positive patients [167]. Early investigations mostly demonstrated reduced adverse effects of drugs encapsulated into liposomes, with a slight superiority in bioavailability. Various liposomal formulations of anticancer agents have been shown to cause fewer adverse effects than free drugs. One classic example is the reduction in cardiotoxicity of doxorubicin, which had limited its frequent use. Doxorubicin in liposomal formulation has shown low cardiotoxicity [168], and with the incorporation of cardioprotective agents, such as quercetin, it has further improved its tolerability [169]. It was also reported that liposomal chemotherapy, despite improved tolerability, showed low efficacy against primary and secondary liver tumors. Testing in human volunteers generally indicated a reduction in adverse effects and improved tolerability of administration but did not ensure superior therapeutic action over other novel drug delivery systems. Numerous drug delivery systems are in different test stages of the drug development process, showing diverging results. PEGylated magnetic niosomes of carboplatin have shown desired characteristics in sustained drug release and improved therapeutic performance. In addition, these niosomes could induce cytotoxicity towards MCF-7, a breast cancer cell line [170]. 

Liposomes can be targeted to the affected tissues by attaching a targeting ligand. Due to the high nutritional demand of rapidly proliferating cancer cells, their uptake of folic acid is very rapid. This strategy has been adopted to deliver liposomes specifically to cancer cells by physically adsorbing folic acid on the liposome surface or by conjugation to the phospholipids incorporated into the lipid bilayer [171,172]. Other nutritional factors are also used for cancer cell-specific delivery, such as pectin [173,174] and mannose [175,176]. Monoclonal antibodies against cancer are the most common type of tumor-targeting ligands. Generally, antibodies are selected for cellular targets overexpressed in cancer cells. Cell membrane proteins (CD133, CD147, and CD44) [177] and membrane receptors such as transferrin receptor [178,179], epidermal growth factor receptor-EGFR [180], human epidermal growth factor receptor 2-HER2 [181], and human B-cell antigen, CD20 [182], are the commonly used antibodies for cancer cells targeting. As cytokines play a key role in the innate and adaptive immune response to cancer, many researchers have added cytokines to targeted liposomes for synergistic tumor cell killing [183]. 

Cell-penetrating peptides (CPP) are another class of ligands employed for tumor cell targeting. TAT, a predominantly hydrophilic CPP, and QLPVM, a more hydrophobic CPP, have been shown to enhance intracellular drug accumulation by more than 10 times in brain tumor cells [184]. 

#### 5.2.4. Topical Drug Delivery

The similarity between cell membranes and liposomes’ structures makes them desirable systems for drug delivery to the skin. In dermatology, liposomes were initially used for smoothening and restoring effects. The barrier function of the stratum corneum was restored using liposomes during the treatment of atopic dry skin, while at the same time, a drug was also transported through the skin layers [185]. On the other hand, the stratum corneum also impedes the permeation of drugs to the deeper skin tissues.

**Figure 4:**
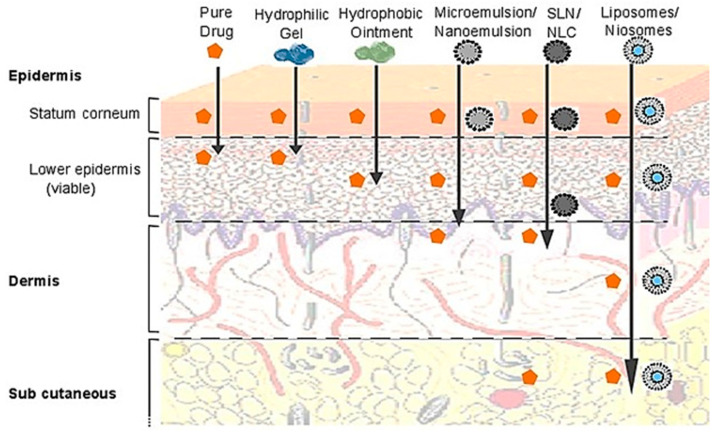
Comparison of penetration of different lipid-based nanocarriers and resulting permeability enhancement across different layers of the skin. Reproduced with modifications (CC BY 4.0) from [186].

Vesicles have been investigated extensively to overcome this barrier with promising results. Niosomes are also attracting attention as effective topical carriers due to improved penetration of vesicles. In a comparative study, enoxacin exhibited increased permeation via niosomal vesicles compared to liposomes composed of dimyristoyl phosphatidylcholine [184]. An overall comparison of lipid-based nanocarrier’s efficiency for drug delivery across the skin is demonstrated in Figure 4.

Cutaneous leishmaniasis is a disease of skin-associated leishmania parasite that is transmitted by the bite of a phlebotomine sand fly. Its treatment is extremely challenging due to the emergence of resistance as the protozoa invade macrophages, bypass killing, and start multiplying inside the macrophages. The effective dose of drugs, mostly different antimonials, is approximately equal to their toxic dose. Passive targeting of infected macrophages in leishmaniosis is possible through the liposomal/niosomal approach. A study of antimony-loaded niosomes in rats revealed their high concentration in hepatocytes after IV injection, suppressing the severity of liver leishmaniasis and proving the ability of niosomes as effective drug carriers [187,188]. 

#### 5.2.5. Oral Drug Delivery 

The application of liposomes in oral therapy is a challenging task owing to the instability problems posed by the GI tract. However, several studies demonstrate the potential of liposomes for increasing the dissolution profile of lipophilic drugs limited by minimal water solubility. In one study, a significant improvement in the oral bioavailability of griseofulvin in albino rats after its encapsulation in niosomes [189]. Recently, diacerein, a BC class II drug, has been encapsulated into niosomes of varying compositions using different surfactants. Sorbitan monolaurate (Span 20), Poloxamer 184, and a mixed surfactant system (a mixture of Span 20 and Poloxamer 184) were evaluated for this purpose. Drug release profiles indicated improved diacerein dissolution profiles, which revealed the suitability of niosomes as oral drug carriers [190]. Further advancing the field, enteric-coated liposomes are prepared by depositing layers of anionic (polyacrylic acid) and cationic (polyallylamine hydrochloride) polyelectrolytes on the liposomes’ surface. The enteric liposome remained stable in the presence of polyelectrolyte coating and led to a 4-fold increase in bioavailability as compared to pure drug or uncoated liposomes [191]. 

#### 5.2.6. Diagnostic and Theragnostic Agents

Theragnostics offer a broad range of applications for vesicles, as they possess a large surface area, allowing covalent and noncovalent functionalization with therapeutic drugs, targeting ligands and hydrophilic polymers. Lipid–quantum dot hybrids loaded with anticancer drugs, lipid–viral nanoparticle hybrids (artificially enveloped adenoviruses), and lipid–polymer hybrid nanoparticles have been used as theragnostic [191,192]. Niosomes are also suggested as diagnostic and therapeutic delivery systems when combined with ultrasound. It was proposed that ultrasound waves can permeabilize the membrane of niosomal vesicles, which permits drug release in a controlled fashion while maintaining the vesicular membrane intact. High-intensity focused ultrasound (HIFU) has been shown to not only promote the accumulation of the drug at the tumor site but also induce targeted drug release by destabilizing the bilayer membrane due to the cavitation effect and induction of hyperthermia [193].

#### 5.2.7. Stimuli-Responsive Liposomes for Targeted Drug Delivery

Stimuli-responsive drug delivery systems are designed to release the payload when they encounter specific stimuli in the body. Encapsulation of drugs and their subsequent release from liposomes is highly dependent on the phospholipid bilayer structure, and a disorder in the bilayer will cause the release of drugs. This property of the liposomes is now widely investigated in the design of liposomes in which the lipid bilayer can be destabilized in the presence of stimuli, and the payload is released immediately [194]. The stimuli may be endogenous or exogenous. Endogenous stimuli include disease-specific biological molecules or changes in tissue microenvironment such as enzymes, hypoxia, redox potential, and pH changes. For example, phospholipase A2 is secreted by tumors and extensively targeted by researchers for tumor-targeted drug delivery. Phospholipase A2 cleaves fatty acids in phospholipids in liposomes bilayer, rapidly releasing small molecule drugs or macromolecules such as antisense polynucleic acids at the tumor site [195]. Liposomes were prepared to contain the anticancer drug paclitaxel and the photosensitizer TPCI for combined chemotherapy–photodynamic therapy. After irradiation, the potency of both drugs was increased by 30 times compared to chemotherapy or thermotherapy alone. In addition, the TPCI could bind the chromatin, causing chromatin aggregation and activating its aggregation-induced emission. Therefore, these systems provide an additional advantage of self-reporting the anticancer effect [196,197].

**Figure 5:**
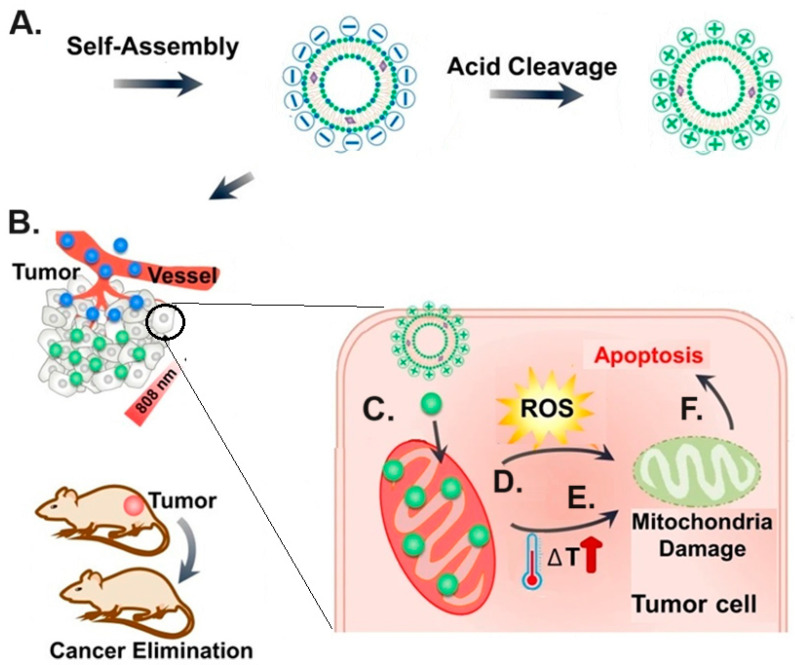
Schematic presentation of cancer-targeting by mitochondria-specific liposomes. Liposomes are formed by self-assembly with indocyanine G (ICG) as a model therapeutic agent (**A**). Liposomes extravasate the blood vessels to reach the acidic tumor microenvironment, where they undergo acid cleavage (**B**). Liposomes enter the cells by micropinocytosis, undergo endosome escape, and enter mitochondria due to carrier-mediated transport (**C**). Irradiation of ICG-loaded liposomes by a laser of 808 nm leads to photodynamic therapy-mediated production of reactive oxygen species (ROS) (**D**) and photothermal therapy (**E**). The resulting mitochondrial damage leads to the induction of apoptosis of the cells (**F**). Reproduced with modifications (CC BY 4.0) from [198].

Acidic pH is a key component of the tumor microenvironment (TME) tissues. For this purpose, acid-cleavable polymers can be attached to the surface of liposomes, and after reaching the tumor site, the polymer is cleaved, and the target cells take up the liposomes. In one study, liposomes were conjugated with mitochondria-specific peptide (G2R) and the acid-cleavable 2,3-dimethyl maleic acid (DA) for targeting intracellular components of cancer cells (Figure 5). The liposomes accumulated at the tumor due to the enhanced permeability and retention (EPR) effect, and DA was cleaved in the acidic TME. Then, liposomes enter the cancer cells by pinocytosis and target the mitochondria due to the presence of G2R peptide. A laser of 808 nm could irradiate the nanoparticles to activate photosensitizer indocyanine G, which caused mitochondrial damage by photodynamic and photothermal therapy, leading to cancer cell apoptosis [198].

Drug release from liposomes can also be induced by applying exogenous stimuli from outside the body. As discussed above, activating a photosensitizer by laser irradiation is also an example of exogenous stimuli. Visible and ultraviolet light have limited penetration into biological tissues, whereas near-infrared (NIR) is usually a preferred choice due to deeper tissue penetration [199,200]. Liposomes loaded with doxorubicin and indocyanine G were used for targeted chemo-thermotherapy of cancer. Liposomes were targeted to cancer by the anti-EGFR peptide. After NIR irradiation, hyperthermia was produced due to the activation of indocyanine G, which killed cancer cells by photothermal effect and destabilized liposomes to induce drug release at elevated temperatures [201]. 

Depending on the melting temperature (Tm) of the lipid hydrocarbon chains, a phase transition from the solid (gel) phase at a temperature lower than Tm to a liquid phase at temperatures above Tm occurs. At a temperature close to the melting point, the bilayer exists in a sol-gel form, and the resulting disordered bilayer releases the payload abruptly. This phenomenon has been used to design thermoresponsive liposomes in which lipid bilayers are disordered when exposed to elevated temperature (above phase transition temperature). NIR irradiation of photosensitizers in photothermal therapy is one example of hyperthermia-induced drug release from thermoresponsive liposomes. Hyperthermia to induce drug release from liposomes can also be achieved by ultrasonication. Due to various advantages, HIFU has emerged as an interesting tool in pharmaceutical sciences, providing excellent control over temperature elevation and ensuring precise control of drug release from thermoresponsive liposomes. The application of HIFU after 10 min of starting the intramuscular infusion of thermoresponsive liposomes leads to a drug concentration 15 times higher than that of the untreated group in a pig model [202]. Local application of high-intensity HIFU can degrade the extracellular matrix, and low-intensity HIFU can lead to extracellular matrix remodeling that facilitates the accumulation of nanoparticles on the target [202]. It can also disrupt biological barriers, such as the BBB, and enhance the penetration of liposomes to the target tissues [202,203,204,205].

Initially, the alternating magnetic field was used to direct the nanoparticles to the target site or induce drug release in the presence of a magnetic moiety [206,207]. As photothermal therapies have shown greater potential for generating hyperthermia, magnetic moieties are now used to track the movement of the multifunctional liposomes in the body by magnetic resonance imaging (MRI), and drug release is induced as a function of photothermal therapy [206,208,209]. Stimuli-responsive liposomes have emerged as the key tool in designing multifunctional drug carriers and are expected to be the most widely studied drug delivery system in the future. 

## 6. Lipid Nanoparticles (LNPs)

Over the past few decades, various delivery systems have been developed for nucleic acid-based vaccines, including viral vectors [210], non-viral vectors, as discussed above [211], and physical methods such as electroporation [212]. Viral vectors, such as adenovirus, lentivirus, and adeno-associated virus (AAV), have shown high transduction efficiency. However, they are associated with safety concerns such as immunogenicity and insertional mutagenesis. Non-viral vectors, such as cationic polymers, lipids, and nanoparticles, are considered safer and more versatile than viral vectors, but their transfection efficiency and safety profiles are highly dependent on their formulation and features, leading to a great deal of variability among the current options available [210,211,212].

LNPs have emerged as one of the most promising non-viral delivery systems for nucleic acid-based vaccines [213]. LNPs are composed of a cationic/ionizable lipid (e.g., DOTAP, DDAB, DOTMA, DODAP, MPPC, DSPE), a helper lipid (e.g., DPPC, DSPC, DOPC), cholesterol, and PEG lipids (e.g., DSPE-PEG, DMPE-PEG) that form a core–shell structure, in which the nucleic acids are encapsulated in the core and the surface is coated with hydrophilic polymers or lipids to improve stability and cellular uptake [214,215]. The LNPs answer several issues that arise from the delivery of nucleic acids, namely by protecting them from degradation from nucleases and for the ability displayed by the LNPs to improve cellular uptake and intracellular release by using the endocytic and lysosomal pathways. In addition, LNPs can be modified to target specific cells or tissues, enhancing their therapeutic effectiveness and, at the same time, improving immune activation and adjuvanticity [216].

Recent clinical trials of mRNA-based vaccines against COVID-19, such as Pfizer/BioNTech’s Comirnaty and Moderna’s mRNA-1273, have demonstrated the safety and efficacy of LNPs as delivery vehicles for nucleic acid-based vaccines. These vaccines have shown high efficacy in preventing COVID-19 infection and severe disease and have been granted emergency use authorization by regulatory agencies worldwide [217,218]. LNPs’ remarkable role in the COVID-19 vaccine is arguably the biggest success story in nanomedicine so far. 

### 6.1. Formulation

The selection and synthesis of lipids are critical in the development of efficient and safe LNPs for nucleic acid-based vaccine delivery. The choice of lipids must consider their biocompatibility, transfection efficiency, stability, and potential to operate as immunoadjuvants. Some commonly used lipids in LNPs include cationic lipids such as DOTAP, DOBAB, and DODAC [219,220], neutral lipids such as cholesterol and phosphatidylcholine, and PEG-modified lipids. The lipids applied in these LNPs can be obtained by chemical synthesis, enzymatic synthesis, or isolated from natural sources [90,221,222]. 

The LNPs help to protect mRNA from degradation and enable the release at the cytoplasmic site. The ratio of ionizable lipid/cationic lipid plays a crucial role in encapsulating or releasing the mRNA. While a phospholipid or helper lipid maintains the outer amphiphilic shell, cholesterol constitutes the outer shell and contributes to molecular recognition of LNPs by apolipoproteins, and finally, PEGylated lipid helps to stabilize the LNPs, preventing aggregation and providing stabilization against opsonization. The choice and composition of lipid components in the structure of LNPs influence the tissue-specific delivery and transfection efficiency. The structure of LNPs can be modified to induce a stronger immune response by promoting the maturation of dendritic cells, neutrophils, and macrophages. A study screened a library of 1080 lipid compositions to find the most suitable lipid for transfection efficiency and reported three LNPs that induced better antigen-specific immune response by subcutaneous injections. These LNPs showed higher levels of Th2 immune response and tumor suppression. The C10 LNPs showed the highest level of transfection efficiency and immune stimulation [223].

The structure–activity analysis of cholesterol analogs revealed a fascinating finding, which plays a crucial role in LNP transfection. Incorporating C-24 alkyl phytosterols into LNPs (eLNPs) enhanced gene transfection. The success of transfection largely depends on the alkyl tail length, the flexibility of the sterol ring, and the polarity resulting from the presence of the -OH group. To maintain high transfection rates, various cholesterol constituents such as 9, 10-Secosteroids (vitamin D derivative), C-24 alkyl steroids (natural phytosterols), and pentacyclic steroids (cholesterol analogs with one additional ring) were evaluated. The results indicated that cholesterol analogs in LNP structures improve their transfection efficiency. These nanoparticles are organized into a core–shell structure, with the core containing the nucleic acid electrostatically complexed with the ionizable lipid, and cholesterol provides structural integrity to the particle [224]. The extra-hepatic delivery of LNPs is a challenging task due to accumulation in the liver and clearance from the body since the LNPs interact with apo-lipoprotein-E and accumulate in the liver. Recently, LNPs were prepared for targeted delivery to the lungs using DLin-MC3-DMA as ionizable lipids, DSPC and DMG-PEG, and β-sitosterol as a cholesterol replacement and observed a higher accumulation in the lungs [225]. LNPs can be prepared for extra-hepatic delivery by using the ionizable lipid C14-3, DOPE, and PEG-lipid, replacing cholesterol with bile acid containing cholesterol replacements such as cholic acid, chenodeoxycholic acid, deoxycholic acid, or lithocholic acid. Bile acid-containing LNPs (BA-LNPs) could reduce delivery to liver cells in vitro and improve the delivery in various other cell types, including T cells, B cells, and epithelial cells. After intravenously or intraperitoneally injection in vivo, BA-LNPs bypassed the liver and accumulated in the spleen at a higher concentration, which is desired to achieve the full therapeutic potential of vaccines, gene therapy, and immunotherapy [226]. 

Transfection efficiency dependence on buffer type and concentration was assessed using sodium citrate (Na citrate), phosphate, and acetate buffers. The result indicated that LNPs containing nominally less active ionizable lipids, formulating them in the presence of high concentrations of Na citrate buffer at pH 4, leads to dramatically improved transfection efficiency both in vitro and in vivo [227].

### 6.2. Mechanisms for Nucleic Acid Delivery 

#### 6.2.1. Cellular Uptake and Intracellular Trafficking

As discussed above, lipid nanoparticles (LNPs) have become a preferred choice for delivering nucleic acid-based vaccines due to their impressive ability to efficiently transport the cargo to targeted cells. This is a significant breakthrough in the development of nucleic acid-based vaccines, as it overcomes one of the primary challenges in the field. LNPs are taken up by cells through various mechanisms, such as receptor-mediated endocytosis, clathrin-mediated endocytosis, caveolae-mediated endocytosis, and macropinocytosis. Once inside the cell, LNPs are transported through the endosomal pathway, where they encounter acidic and hydrolytic environments that could potentially degrade the cargo [228,229].

#### 6.2.2. Endosomal Escape and Cytoplasmic Release

Endosomal escape is a critical step in the intracellular delivery of nucleic acids by LNPs, as it is crucial to ensure therapeutic effect. To escape the endosomal pathway, LNPs are developed according to different strategies to perform the proton sponge effect, membrane fusion, or lysosomal disruption. Once released into the cytoplasm, the nucleic acids can then be further transported to the nucleus, where they can be transcribed or translated [230,231].

#### 6.2.3. Mechanisms of Gene Expression: Transcription, Translation, and Antigen Presentation

The efficient delivery of nucleic acids by LNPs is required to induce gene expression, which is critical for generating an immune response in nucleic acid-based vaccines. Transcription of the delivered nucleic acid can result in the production of mRNA, which can then be translated into proteins. Alternatively, the delivered nucleic acid can be directly translated into proteins through mechanisms such as cap-independent translation. The generated proteins can then be presented to the immune system, resulting in the induction of an immune response [232,233]. LNPs play a crucial role in the efficient delivery of nucleic acid-based vaccines to target cells. The cellular uptake, intracellular trafficking, endosomal escape, and cytoplasmic release of LNPs are critical steps in this process. The efficient delivery of nucleic acids by LNPs can result in the induction of gene expression and the generation of an immune response.

### 6.3. Clinical Development of LNP-Based Nucleic Acid Vaccines

The development of LNP-based nucleic acid vaccines has been a rapidly evolving field in recent years, with several candidates moving into clinical trials. This section discusses the various phases of clinical trials for nucleic acid-based vaccines and the safety, immunogenicity, and efficacy endpoints evaluated in these trials.

Phase I trials are typically small-scale studies that aim to evaluate the safety and tolerability of a vaccine candidate in healthy volunteers. These trials also look at the pharmacokinetics and pharmacodynamics of the vaccine, including the immune response elicited by the vaccine. Phase II trials are larger-scale studies that evaluate the safety and efficacy of the vaccine candidate in a larger population. Phase III trials are even larger studies that further evaluate the safety and efficacy of the vaccine candidate in a larger population and can include a placebo control group. Finally, Phase IV trials are post-approval studies that evaluate the long-term safety and effectiveness of the vaccine [234,235].

One of the most successful examples of an LNP-based nucleic acid vaccine is the mRNA COVID-19 vaccine developed by Pfizer-BioNTech and Moderna. These vaccines, which vehiculate mRNA encoding the spike protein of the SARS-CoV-2 virus to human cells within LNPs, generate an immune response and protect against SARS-CoV-2-induced disease. Both vaccines have demonstrated high efficacy rates in clinical trials and received emergency use authorization from regulatory agencies worldwide [236,237].

Other LNP-based nucleic acid vaccines in clinical development include DNA vaccines for various infectious diseases and cancer. Inovio Pharmaceuticals has developed a DNA vaccine for COVID-19 that uses a LNP delivery system. The vaccine has shown promising results in early clinical trials, with high antibody and T-cell responses in vaccinated individuals [238]. Another DNA vaccine in clinical development is VGX-3100, developed by Inovio Pharmaceuticals for the treatment of cervical dysplasia caused by human papillomavirus. The vaccine uses an LNP delivery system to deliver DNA-encoding antigens of the virus to the cells of the cervix, inducing an immune response [239].

As with all vaccines, as well as all medicines, regulatory approval is a critical step in the development and commercialization of LNP-based nucleic acid vaccines. Regulatory agencies such as the U.S. Food and Drug Administration (FDA) and the European Medicines Agency (EMA) have developed specific guidelines for the development and approval of nucleic acid-based vaccines [240,241]. In addition to regulatory considerations, future endeavors for LNP-based vaccines include the development of vaccines for a wider range of infectious diseases and cancer. Their versatility, based on the ability to fine-tune a lot of their properties, combined with their efficacy, makes LNP-based delivery systems an attractive option for delivering nucleic acid-based vaccines, and ongoing research is likely to uncover new applications for these technologies [242,243,244,245]. In conclusion, LNP-based nucleic acid vaccines represent a promising and rapidly evolving field in vaccine development. The success of the mRNA COVID-19 vaccines has brought this technology to the forefront of public attention, but ongoing research is likely to uncover new applications and opportunities for these innovative vaccine delivery systems.

## 7. Conclusions

Over the past years, lipid-based nanocarriers have found diverse applications in drug delivery, and the number of lipid-based nanoformulations approved for clinical use is increasing. Lipid-based nanoparticles have opened the possibility of developing a safe, cheap, and promising approach for drug delivery through several routes of administration. Lipid nanoparticles can be made with a wide range of structural features that, in turn, provide the opportunity to encapsulate small and macromolecular agents, control spatial and temporal drug release, and adjust the permeability across biological barriers. Pharmaceutical manufacturers are increasingly focused on developing multifunctional and advanced drug delivery systems that can precisely control the release of medication. The successful implementation of these lipid-based delivery systems depends on scaling up the technology from the laboratory to clinical application. To ensure the suitability of lipid-based carriers for human use, it is important to consider factors such as biodistribution, bioavailability, toxicology, and formulation stability. It is widely anticipated that these lipid-based delivery systems will play a significant role in the global pharmaceutical market in the decades to come. A growing number of lipid-based nanoformulations have already reached clinical application, with promising results in enhancing therapeutic efficacy and targeting, underscoring their potential to transform the delivery of future treatments across various medical fields.
